# ﻿Contribution to the Chinese *Intybia* Pascoe, 1866 (Coleoptera, Melyridae, Malachiinae), with descriptions of two new species groups and one new species

**DOI:** 10.3897/zookeys.1202.115935

**Published:** 2024-05-27

**Authors:** Yuqi Wang, Zhiqiang Li, Ping You, Zhenhua Liu

**Affiliations:** 1 Guangdong Key Laboratory of Animal Conservation and Resource Utilization, Guangdong Public Laboratory of Wild Animal Conservation and Utilization, Institute of Zoology, Guangdong Academy of Sciences, Guangzhou 510260, China Institute of Zoology, Guangdong Academy of Sciences Guangzhou China; 2 College of Life Science, Shaanxi Normal University, Xi’an, 710062, China Shaanxi Normal University Xi’an China

**Keywords:** Apalochrini, Cleroidea, Hainan, key, redescription, taxonomy

## Abstract

A contribution to the knowledge of the malachiine genus *Intybia* Pascoe, 1866 from China is given. Two new species groups: *Intybiaklapperichi* group and *Intybiaeversi* group are defined and described. A new species, *Intybiahainanensis* Wang & Liu, **sp. nov.**, of the *Intybiaklapperichi* group is described from Hainan Province. *Intybiaerectodentatus* (Wittmer, 1982) and *Intybiaconcha* Asano, 2015 are redescribed based on new materials collected in mainland China. A key to species groups of the genus *Intybia* Pascoe, 1866 in China is provided.

## ﻿Introduction

The genus *Intybia* Pascoe, 1866 belongs to the tribe Apalochrini of malachiine Melyridae, which is represented by about 170 species from the Asiatic region, making it one of the most diverse genera within the tribe Apalochrini (Wittmer 1997; Plonski 2013; Tshernyshev 2016; Ikeda and Yoshitomi 2017). It is characterized by the following characters: antenna with scape and antennomere 3 dilated and modified in the male; fore leg with tarsus and tibia simple in the male, tarsal formula 5–5–5; and pronotum without bead along lateral or posterior margins. Initially, most species of Apalochrini were included in *Laius* Guérin-Méneville, 1830 (Macleay 1872; Blackburn 1888; Lea 1909). Champion (1921) treated *Intybia* as a subgenus of *Laius*, until Evers (1994) re-diagnosed *Laius* and recognized *Intybia* as an independent genus. This treatment was followed by most researchers, and subsequently, a considerable number of species were transferred from *Laius* to *Intybia* (Wittmer 1995, 1997, 1999; Yoshitomi and Lee 2010; Plonski 2013, 2014a, 2014b; Plonski and Geiser 2014). Plonski and Geiser (2014) and Plonski (2016) divided *Intybia* into 11 species groups mainly based on colouration. A new subgenus, Intybia (Protolaius) Tshernyshev, 2020 was described for several species of the *Intybialombokana* group based on male characters of the fore femora (Tshernyshev 2020). Tshernyshev (2015) established a new genus, *Troglointybia* Tshernyshev, 2015, for a few species of *Intybia* with a sculptured head; more species from Southeast Asia were transferred to this genus by Tshernyshev (2016). Plonski (2016) suggested eyestalks as the autapomorphy of *Troglointybia* and transferred most species back to *Intybia*, leaving only four species in *Troglointybia*.

Research on the genus *Intybia* is relatively abundant in some Asian regions, including Japan (Ikeda and Yoshitomi 2017; Asano 2021), Philippines (Plonski 2014b; Tshernyshev 2016), the Himalaya region (Tshernyshev 2015) and Indonesia (Wittmer 1995). In China, there are approximately 28 recorded species of the genus *Intybia*, most of which were described by Wittmer (1955, 1956, 1982, 1995, 1996, 1997) and Pic (1907, 1910, 1919, 1921, 1927). According to Plonski (2016), most species of Chinese *Intybia* can be assigned to five species groups: *Intybiaguttata* group, *Intybiapelegrini* group, *Intybiapicta* group, *Intybiarubrithorax* group and *Intybiavenusta* group. While *Intybiaklapperichi* (Hicker, 1949), *Intybiaerectodentatus* (Wittmer, 1982), *Intybiaevers*i (Hicker, 1949) and *Intybiaconcha* Asano, 2015 are not assignable to any species group described by Plonski (2016) based on specimens and original descriptions; thus, two new species groups are described here. One new species collected from Hainan Province, similar to *Intybiaklapperichi* and *Intybiaerectodentatus*, is also described.

## ﻿Material and methods

*Intybia* specimens involved in this study are deposited in the following institutions: **IZGAS**–Institute of Zoology, Guangdong Academy of Sciences, Guangzhou, China; **ZFMK**–Zoologisches Forschungsmuseum Alexander Koenig, Bonn, Germany.

Specimens for dissections were cleared in a 10% solution of KOH for about 10 h at room temperature. The abdomen with the aedeagus was transferred to a cavity slide using fine forceps and the aedeagus was separated from the abdomen using a hooked, fine dissecting needle. Specimens were mounted on cards with white emulsion glue. Genitalia and terminal abdominal segments are preserved in genitalia vials with glycerol.

The habitus images were captured using a Canon 7D DSLR camera, Canon MP–E 65 mm macro lens and Mitutoyo 5× objective lens, mounted on a WeMacro Focus Stacking Rail, with Helicon Remote 3.9.10 and WeMacro software for focus stacking. Male genitalia were photographed using a Zeiss AxioCam HRc digital camera mounted on a Zeiss AX10 compound microscope with the Axio Vision SE64 4.8 software. Layered images of the male genitalia were stacked in Helicon Focus software and edited in Photoshop CC 2022.

The morphological terms used in this paper follow Lawrence and Ślipiński (2013). Measurements were made as follows: body length—from the apical edge of the clypeus to the apex of the elytra; pronotal length—median line from the anterior margin to the posterior margin; pronotal width—maximum width of the pronotum; elytral length—from the base of the scutellum to the elytral apex along the suture; elytral width—maximum width across the elytra.

## ﻿Taxonomy

**Melyridae Leach, 1815**

**Malachiinae Fleming, 1821**

***Intybiaklapperichi* group**

**Figure 1.**
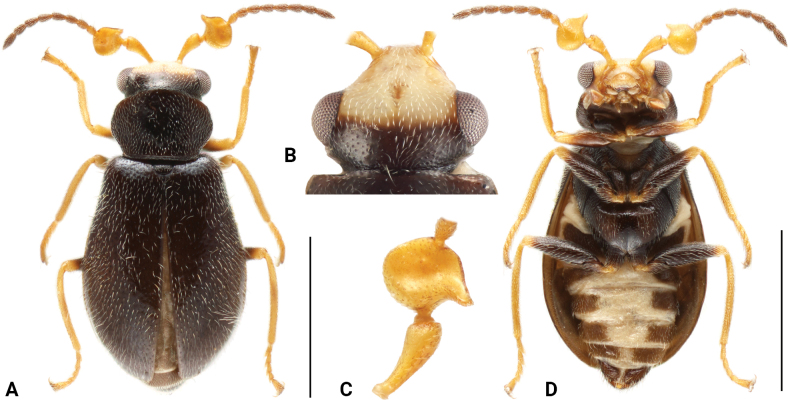
*Intybiahainanensis* sp. nov., holotype **A** habitus, dorsal view **B** head, dorsal view **C** basal antennoeres, dorsal view **D** habitus, ventral view. Scale bars: 1 mm (**A, B**).

**Diagnosis.** Body size small (less than 2.5 mm). Head black with anterior area yellow, pronotum and elytra entirely black; antenna with basal three segments yellow, antennomeres 4–11 brownish to dark brown; legs yellow with basal 4/5 of femora brown to black (Figs 1A, D, 3A, B). Vestiture of short whitish setae. Dorsal surface without distinct punctuation. Antennomere 3 suboval, with projection along inner edge (Figs 1C, 3D). Pronotum transverse, slightly constricted at base.

**Included species.***Intybiaerectodentatus* (Wittmer, 1982), *Intybiahainanensis* Wang & Liu, sp. nov., *Intybiaklapperichi* (Hicker, 1949).

**Distribution.** Only known from China: Fujian, Guangdong, Hainan, Taiwan.

### ﻿ Intybia hainanensis

Taxon classificationAnimaliaColeopteraMelyridae

Wang & Liu sp. nov.

191E314D-1C59-5BFF-8E30-99C9B66BB1D5

https://zoobank.org/C25966F5-97F1-4B7F-9278-6A67E63296B0

Figs 1 , 2A–C

#### Type material.

***Holotype***: China • ♂; Hainan, Ledong, Jianfengling; 18.731273°N, 108.873082°E; 17 Mar. 2021; Yuchen Zhao leg; IZGAS. ***Paratype***: • 1 ♂; same data as for holotype.

#### Diagnosis.

This species is similar to *Intybiaerectodentatus* (Wittmer, 1982) and *Intybiaklapperichi* (Hicker, 1949) in body shape and colouration. It can be easily recognized by the shape of antennomere 3 (Fig. 1C), whose inner protrusion is pointed apically (blunt in the other two species).

#### Description.

**Male.** Length 2.0–2.1 mm. Head yellow, with areas behind middle of eyes black on dorsal surface, frons with darker median spot (Fig. 1B); ventral surface with gular area more? or brownish. Mandibles yellow with black apex, maxillary palps and labial palps brownish. Antennae with antennomeres 1–4 yellow, remaining 7 segments brown. Pronotum and elytra black. Legs yellow with basal 4/5 of femora and apex of trochanters dark brown to black. Prosternum dark brown, mesoventrite and metaventrite black; abdominal ventrites dark brown with medial areas of ventrite 2–5 yellow (Fig. 1A, D). Vestiture of short yellowish setae.

Head widest across eyes, nearly as wide as pronotum; dorsal surface flat, covered with dense short setae, clypeus divided into sclerotized postclypeus and membranous anteclypeus; frons weakly depressed, slightly constricted in front of eyes. Eyes relatively large and laterally protruding. Antenna with 11 segments; scape subtriangular, elongated with slightly enlarged apex; antennomere 3 enlarged and suboval, inner edge with inwardly twisted projections; antennomeres 4–11 covered with short white setae. Labrum transverse, dorsal surface convex. Mandibles bidentate apically, slightly blunt, inner margin straight. Maxillary palps with 4 segments, last segment enlarged and obliquely truncated; labial palps 3 with segments, segment 1 very short, segment 3 conical.

Prothorax transverse; pronotum about 0.7 times as long as wide, widest at about basal third, distinctly constricted at base (Fig. 1A); anterior margin rounded and posterior margin nearly straight. Surface covered with short white setae. Prosternum short, protrochantins exposed. Procoxal cavities transverse and nearly contiguous, posteriorly open; procoxae enlarged and distinct protruding. Scutellum transverse with posterior margin slightly rounded.

Elytra about 1.4 times as long as wide, pear-shaped, widest at about anterior third; humeri rounded and slightly elevated. Surface covered with short white setae. Mesoventrite and metaventrite covered with white setae. Mesoventrite short and subtriangular; mesocoxal cavities large, contiguous at middle, laterally open to mesepimeron; mesocoxae subtriangular, apex slightly swollen, distinctly projecting. Metaventrite enlarged; discrimen distinct, not extending to center, lateral areas with punctures; metanepisternum broad, narrowed posteriorly. Metacoxae transverse, subtriangular, sharply narrowed beside trochanter.

Legs (Fig. 1C) slender, femora slightly enlarged at middle, covered with dense short setae; tibiae slender, with denser setae than femora, which is even denser on inner surface of fore tibiae. Tarsal formula 5–5–5; tarsomeres 3 and 4 slightly shorter than tarsomeres 1 and 2; tarsomere 5 longest, with pair of symmetrical small claws and membranous appendages.

**Figure 2.**
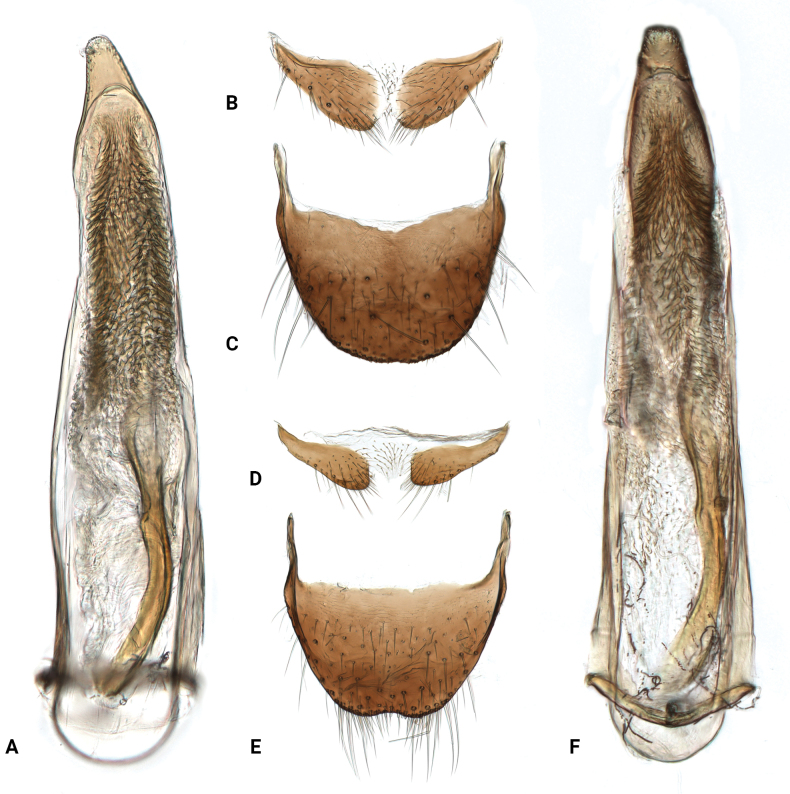
*Intybiahainanensis* sp. nov., paratype (**A–C**) and *Intybiaklapperichi* (Hicker, 1949) (**D–F**) **A, F** aedeagus, dorsal view **B, D** sternite VIII **C, E** tergite VIII.

Abdomen with 6 freely movable ventrites; ventrite 1 divided by metacoxae; ventrites 2–4 subequal in length and gradually decreasing in width. Ventrites 2–6 covered with white to light yellow setae on both sides, with longer setae on the last 2 ventrites. Tergite VIII (Fig. 2C) with apical margin broadly rounded, covered with sparse setae; sternite VIII (Fig. 2B) weakly connected at middle. Aedeagus (Fig. 2A) slender, narrowly rounded apically; endophallus with longitudinal sclerite about half as long as penis, curved near base, subapical area with numerous spinules.

**Female.** Unknown.

#### Etymology.

The species name is derived from the province Hainan, where the type specimens were collected.

#### Distribution.

Hainan.

### ﻿ Intybia klapperichi

Taxon classificationAnimaliaColeopteraMelyridae

(Hicker, 1949)

849C6871-92A3-560D-AEEB-007277B6C377

Figs 2D–F , 3

#### Material examined.

***Holotype***: China • ♂; Fujian, Kwangtseh; 1 Sept. 1937; J. Klapperich leg.; ZFMK-COL-1000188.

#### Other materials.

China • 2 ♂, 4 ♀; Guangdong, Shaoguan City, Ruyuan, Nanling National Forest Park; 24.88487°N, 113.03585°E; 9 May 2023; Zhenhua Liu, Yuqi Wang and Liye Wei leg.; net sweeping; IZGAS.

#### Diagnosis.

**Figure 3.**
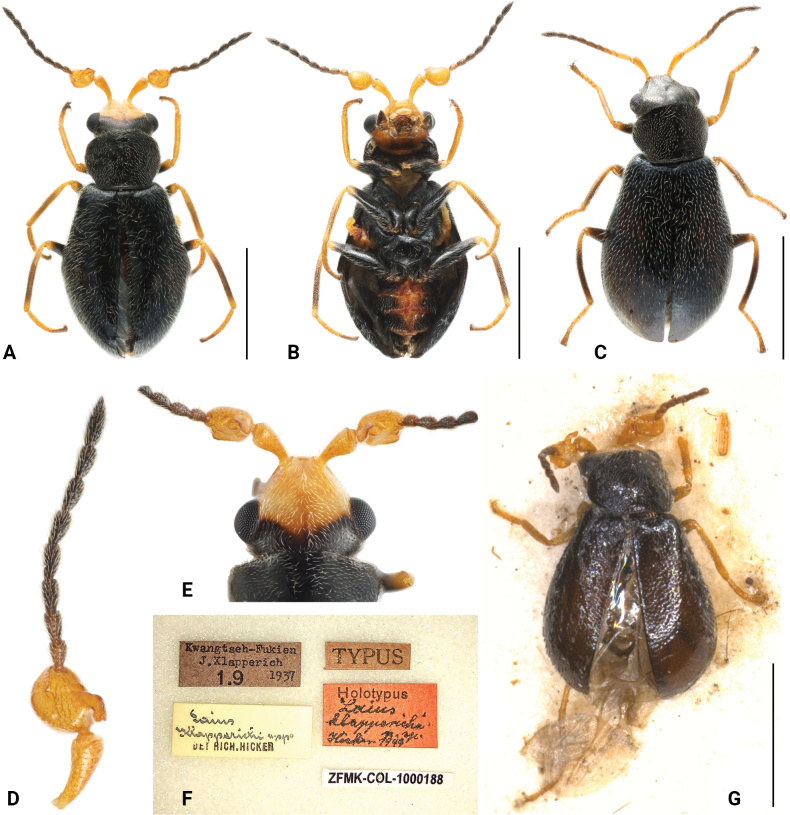
*Intybiaklapperichi* (Hicker, 1949) **A** male habitus, dorsal view **B** male habitus, ventral view **C** female habitus, dorsal view **D** male antenna, dorsal view **E** head, dorsal view **F** holotype, labels **G** holotype, dorsal view. Scale bars: 1 mm (**A–C, G**).

It can be distinguished from the other two species within the species group by shape of antennomere 3 (Fig. 3D), with inner protrusion blunt apically and bent toward posterior (inner protrusion pointed apically in *Intybiahainanensis* and bent toward anterior in *Intybiaerectodentatus*).

#### Redescription.

**Male.** Length 2.0–2.4 mm. Head yellow, with areas behind middle of eyes black on dorsal surface (Fig. 1A, G), frons with median area darker (Fig. 3E); ventral surface with gular area dark brown. Mandibles yellow with black apex; maxillary palps and labial palps dark brown. Antennae with scape and antennomere 3 light yellow, antennomeres 4–11 brown to black (Fig. 3D). Pronotum and elytra black. Legs with coxae, trochanters and femora mostly black; apex of tarsi, middle areas of middle and hind tibiae brownish to dark brown; remaining part of tibiae and tarsi, joints of coxae and trochanters yellow (Fig. 3B). Prosternum, mesoventrite and metaventrite black; abdominal ventrites black with medial areas yellow to orange. Vestiture of dense white setae.

Head widest across eyes, nearly as wide as pronotum; dorsal surface flat, covered with short setae; clypeus divided into sclerotized postclypeus and membranous anteclypeus; frons slightly constricted in front of eyes. Eyes large and laterally protruding. Antenna with 11 segments; scape elongated and subtriangular; antennomere 3 suboval, inner margin with a hammer-shaped projection twisting towards posterior (Fig. 3D); antennomeres 4–11 densely covered with setae. Labrum transverse, anterior margin strongly arched. Maxillary palps with 4 segments, last segment enlarged and obliquely truncate; labial palps with 3 segments, terminal segment conical.

Prothorax transverse; pronotum about 0.7 times as long as wide, widest at about half; margins smooth, without distinct angles; anterior margin evenly arched, posterior margin nearly straight. Surface densely covered with white short setae. Prosternum short, protrochantin exposed. Procoxal cavities transverse and contiguous at middle; procoxae enlarged, subtriangular. Scutellum subtrapezoid, posterior margin nearly truncated.

Elytra about 1.4 times as long as wide, widest at about anterior third, lateral margins slightly curved; humeri slightly elevated. Surface densely covered with white setae, longer than those on pronotum, punctation indistinct. Mesoventrite transverse and subtriangular; mesocoxal cavities contiguous at middle, laterally open to mesepimeron; mesocoxae enlarged and subtriangular, distinctly projecting, trochantins exposed. Metaventrite transverse, slightly swollen, metanepisternum broad at base, narrowed posteriorly. Metacoxae transverse, subtriangular, sharply narrowed beside trochanter.

Legs (Fig. 3B) slender, femora slightly enlarged at middle, covered with dense short setae; tibiae slender, with denser setae than femora, which is even denser on inner surface of fore tibiae. Tarsal formula 5–5–5, terminal tarsomere with pair of symmetrical small claws and membranous appendages.

Abdomen with 6 freely movable ventrites. Ventrites with long setae on the sides, longer setae on ventrites 4–6. Tergite VIII with posterior margin emarginate (Fig. 2E), covered with sparse setae, denser along apical margin; sternite VIII narrowly weakly connected at middle (Fig. 2D). Aedeagus (Fig. 2F) slender, apex narrowly rounded; endophallus with longitudinal sclerite, about half as long as penis; subapical area with numerous spinules.

**Female.** Similar to male in body shape and colouration (Fig. 3C), but with head entirely black. Antenna with scape only slightly dilated apically; antennomere 3 simple, rectangular. Fore tarsi distinctly longer than those in male.

#### Distribution.

Fujian, Guangdong.

***Intybiaeversi* species group**

**Figure 4.**
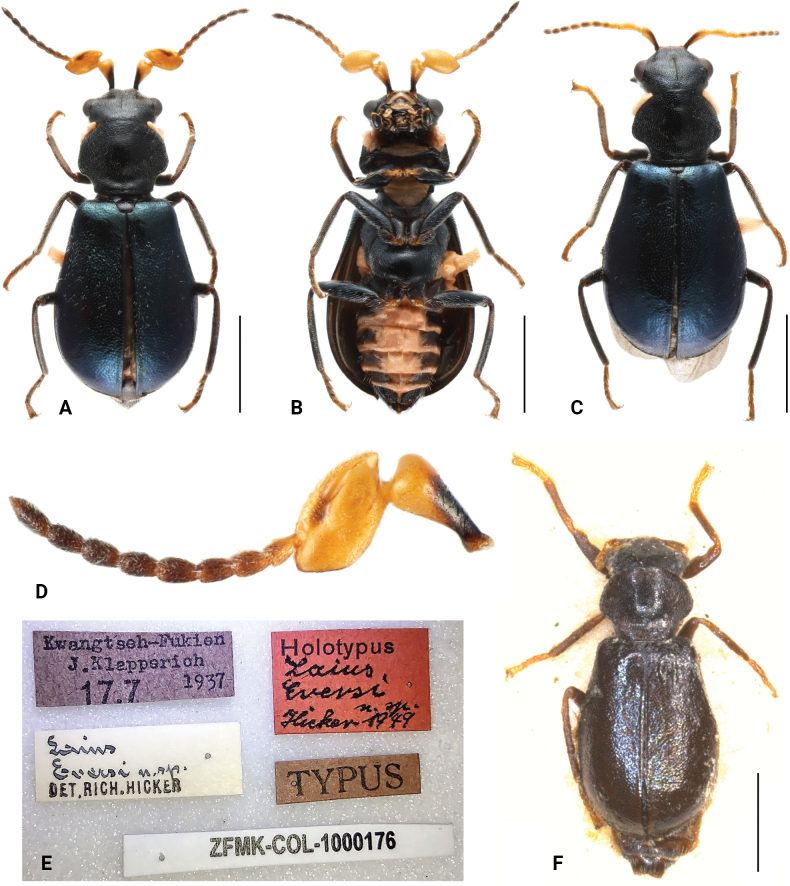
*Intybiaeversi* (Hicker, 1949) **A** male habitus, dorsal view **B** male habitus, ventral view **C** female habitus, dorsal view **D** male antenna, dorsal view **E** holotype, labels **F** holotype, dorsal view. Scale bars: 1 mm (**A–C, F**).

**Diagnosis.** Head, pronotum, scutellum and elytra black, elytra with metallic blue luster (Fig. 4A–C, F). Vestiture of dense short setae. Head with midcranial suture. Pronotum strongly constricted posteriorly, distinctly shorter than elytra at base. Elytra only with dense fine punctures at center, remaining areas nearly smooth. This species group resembles the *Intybialombokana* group (Plonski, 2015) in colouration, but can be easily distinguished by the simple vestiture, punctation on the elytra and shape of the pronotum.

**Remarks.**Tshernyshev (2020) established the subgenus Protolaius for *Intybialombokana* (Pic, 1910) and *Intybiaschillhammeri* (Wittmer, 1966) of the *Intybialombokana* group, mainly based on the excavate femora in the male, which is absent in *Intybiaconcha*.

**Included species.***Intybiaconcha* Asano, 2015, *Intybiaeversi* (Hicker, 1949).

**Distribution.** Only known from China (Guangdong, Jiangsu, Shaanxi, Taiwan).

### ﻿ Intybia eversi

Taxon classificationAnimaliaColeopteraMelyridae

(Hicker, 1949)

BC5FDEC7-CDF1-5BE8-B756-92D01308EA5B

Figs 4 , 5

#### Material examined.

***Holotype***: China • ♂; Fujian, Kwangtseh; 17 July 1937; J. Klapperich leg.; ZFMK-COL-1000176.

#### Other materials.

China • 1 ♂, 1 ♀; Zhejiang, Shaoxing City, Yuecheng District, Fusheng Town; 29.94070°N, 120.73935°E; 14 July 2020; Yuchen Zhao leg.; net sweeping; IZGAS; • 1 ♂; Hubei, Huanggang City, Macheng Aimenguan; 565 m; 31.3856863°N, 115.3231569°E; Fei Ye and Yuqi Wang leg.; IZGAS • 3 ♀; Guangdong, Shaoguan City, Nanling National Forest Park first peak; 24.91971°N, 112.974713°E; 1541 m; 24 Aug.–28 Sept. 2022; Wenfeng Li and Ruonan Zhang leg.; malaise trap; IZGAS.

#### Diagnosis.

This species can be distinguished by its scape with a dark stripe along the inner margin (Fig. 4D), head with a midcranial suture and fine punctations on the elytra. Compared to *Intybiaconcha*, antennomere 3 of this species has a smoother edge, presenting a more regular spindle shape, with the outer margin slightly blunter than the inner margin.

#### Redescription.

**Male.** Length 2.7–3.2 mm. Head black, with areas between antennal insertions and anteclypeus yellowish; labrum black at base, apical area yellow, maxillary palps with apex of terminal segment dark yellow. Antenna with basal 3 segments yellow, base of scape black, antennomere with dark stripe along anterior edge on dorsal surface; antennomere 4–11 brownish to dark brown (Fig. 4D). Pronotum black, elytra iridescent dark blue with metallic lustre. Legs deep brown to black with tarsi light brown. Ventral side black, with medial areas and posterior margins of abdominal ventrites 2–5 orange, ventrites 2 to 5 extending yellow regions laterally (Fig. 4B). Vestiture of short white setae.

Head widest across eyes, narrower than pronotum; dorsal surface flat, with distinct midcranial suture; frons slightly constricted in front of eyes; clypeus divided into sclerotized postclypeus and membranous anteclypeus. Eyes large and conspicuously prominent. Antenna with 11 segments; scape subtriangular, with apex distinctly dilated; antennomere 3 strongly transverse, with large transverse concavity, inner edge slightly pointed; antennomeres 4–11 covered with dense white short setae. Labrum transverse and large, nearly semicircular, apical margins strongly arched. Maxillary palps with 4 segments with terminal segment dilated and obliquely truncate; labial palps with 3 segments with terminal segments conical.

Pronotum slightly transverse, about 0.8 times as long as wide, widest at about basal third; lateral margins strongly constricted at base, anterior margin curved, posterior margin almost straight (Fig. 4A, F); disc convex dorsally, slightly depressed posteriorly. Surface nearly smooth, without distinct punctuation; densely covered with short setae. Prosternum short, protrochantins exposed. Procoxal cavities strongly transverse, contiguous at middle; procoxae enlarged and ventrally protruding. Scutellum subtrapezoidal, posterior margin truncate; surface covered with sparse short setae.

Elytra about 1.2 times as long as wide, widest at about apical third, ovoid; humeri slightly elevated; surface covered with dense fine punctures at middle, remaining areas nearly smooth, covered with dense short setae. Mesoventrite elongated; mesocoxal cavities contiguous at middle, laterally open to mesepimeron; Mesocoxae large and triangular, ventrally protruding. Metaventrite slightly swollen, with short discrimen; metacoxae transverse, subtriangular, sharply narrowed beside trochanters.

Legs slender, femora slightly swollen, covered with white short setae; tibiae slender, with denser setae than femora, which is even denser on inner surface of fore tibiae. Tarsal formula 5–5–5, front tarsi distinctly shorter; terminal tarsomere with pair of symmetrical small claws and membranous appendages underneath.

**Figure 5.**
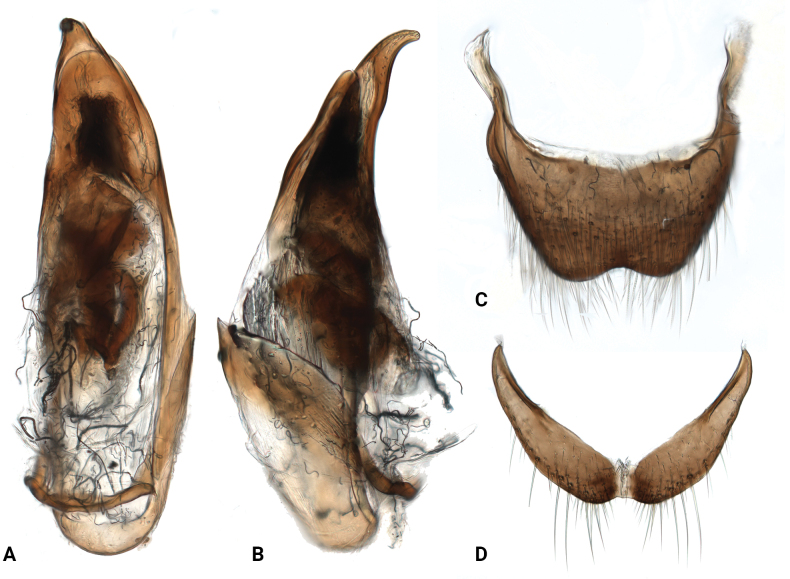
*Intybiaeversi* (Hicker, 1949), male genitalia **A** aedeagus, dorsal view **B** aedeagus, lateral view **C** tergite VIII **D** sternite VIII.

Abdomen with 6 freely movable ventrites; ventrites 1–4 subequal in length, longer than apical 2 segments, ventrites 4–6 gradually narrowed to apex. Tergite VIII (Fig. 5C) subtrapezoid, with pair of moderately long and curved posterior struts, posterior margin triangularly emarginated; sternite VIII (Fig. 5D) transverse, weakly connected at middle. Aedeagus broad at middle (Fig. 5A), apex acute and curved (Fig. 5B); endophallus with 4 broad and curved sclerites at middle, and 4 small spine–shaped sclerites next to them, 3 at basal and 1 at apical, subapical area with a few thick spinules.

**Female.** Similar to male in body shape and colouration (Fig. 4C), but antenna with scape only slightly dilated apically, antennomere 3 simple and elongated, front tarsi longer.

#### Distribution.

Fujian, Guangdong.

### ﻿Key to species groups of the genus *Intybia* Pascoe in China

On the basis of species group designations proposed by Plonski (2016), a key to species groups of *Intybia* in China is provided below:

**Table:** 

1	Elytra monochrome, without spot or stripe	**2**
–	Elytra not monochrome, with whitish, yellowish or orange spots or stripes	**4**
2	Thorax orange-red; elytra dark blue with metallic luster, with dense and coarse punctuation	***Intybiarubrithorax* group**
–	Thorax black; elytra black, with or without metallic blue luster, without distinct. punctation	**3**
3	Elytra black with metallic blue luster, head with frons black; pronotum strongly constricted at base; body length more than 3.5 mm	***Intybiaeversi* group**
–	Elytra black without metallic luster, head with frons yellow; pronotum gradually constricted to base; body length less than 2.5 mm	***Intybiaklapperichi* group**
4	Thorax orange-red; elytra with two transverse orange stripes mixed with white, which are connected along the suture	***Intybiavenusta* group**
–	Thorax black; elytra with one transverse stripe or three whitish spots	**5**
5	Elytra black without metallic luster, with three whitish spots, one at about anterior third and two subapical	***Intybiaguttata* group**
–	Elytra black, sometimes with metallic luster, with one yellow to orange transverse stripe before middle, sometimes continuous across suture	***Intybiapelegrini* group**

## Supplementary Material

XML Treatment for Intybia hainanensis

XML Treatment for Intybia klapperichi

XML Treatment for Intybia eversi

## ﻿Additional information

### Author ORCIDs

Yuqi Wang https://orcid.org/0009-0002-0311-8445

Zhiqiang Li https://orcid.org/0000-0003-0064-7165

Ping You https://orcid.org/0000-0002-2913-1544

Zhenhua Liu https://orcid.org/0000-0002-2739-3305

### Data availability

All of the data that support the findings of this study are available in the main text.
